# Biophysical and Biochemical Outcomes of *Chlamydia pneumoniae* Infection Promotes Pro-atherogenic Matrix Microenvironment

**DOI:** 10.3389/fmicb.2016.01287

**Published:** 2016-08-17

**Authors:** Shankar J. Evani, Shatha F. Dallo, Anand K. Ramasubramanian

**Affiliations:** ^1^Department of Biomedical Engineering, University of Texas at San Antonio, San AntonioTX, USA; ^2^South Texas Center for Emerging Infectious Diseases, San AntonioTX, USA

**Keywords:** 3D, intima, collagen, endothelial dysfunction, *Chlamydia pneumoniae*, stiffness, atherosclerosis

## Abstract

Multiple studies support the hypothesis that infectious agents may be involved in the pathogenesis of atherosclerosis. *Chlamydia pneumoniae* is strongly implicated in atherosclerosis, but the precise role has been underestimated and poorly understood due to the complexity of the disease process. In this work, we test the hypothesis that *C. pneumoniae*-infected macrophages lodged in the subendothelial matrix contribute to atherogenesis through pro-inflammatory factors and by cell-matrix interactions. To test this hypothesis, we used a 3D infection model with freshly isolated PBMC infected with live *C. pneumoniae* and chlamydial antigens encapsulated in a collagen matrix, and analyzed the inflammatory responses over 7 days. We observed that infection significantly upregulates the secretion of cytokines TNF-α, IL-1β, IL-8, MCP-1, MMP, oxidative stress, transendothelial permeability, and LDL uptake. We also observed that infected macrophages form clusters, and substantially modify the microstructure and mechanical properties of the extracellular matrix to an atherogenic phenotype. Together, our data demonstrates that *C. pneumoniae*-infection drives a low-grade, sustained inflammation that may predispose in the transformation to atherosclerotic foci.

## Introduction

In addition to the well-known risk factors for atherosclerosis, recent evidence convincingly suggests that infectious agents may be involved in the development of atherosclerosis (Sessa et al., 2014). Of interest, the intracellular bacterium, *C. pneumoniae*, has been implicated as a risk factor for atherosclerosis based on several *in vitro* cell culture, seroepidemiological, histopathological, animal models, and limited clinical intervention studies (Campbell and Kuo, 2004; Roulis et al., 2013). Interestingly, antibiotic trials in murine and rabbit models have shown that acceleration of atherosclerosis is greatly reduced or eliminated if antibiotics are administered shortly after *C. pneumoniae* inoculation of lung, but not if given much later (Fong et al., 2002). Similar observations were made in clinical trials where short-term treatment of atherosclerotic diseases with antibiotics, including roxithromycin, gave significant decrease in aortic aneurysm growth, carotid artery thickness, peripheral vascular disease symptoms and restenosis (Grayston, 2003), but was not effective in the prevention of an advanced, acute coronary event (Grayston et al., 2005). These observations underscore the need to understand the mechanistic contribution of *C. pneumonia*e-infection to different stages of atherosclerosis for interpreting the results of clinical trials, and in designing new viable treatment strategies (Grayston et al., 2015).

*Chlamydia pneumoniae* is an obligate intracellular bacterium associated with community acquired pneumonia, and is ubiquitous in general population. The infectious elementary body (EB) enters the host cell, replicates inside a self-formed vacuole or inclusion, and converts to non-replicating reticulate body (RB), which differentiates back to EB. At the end of the infection cycle, the EBs are released to infect other susceptible host cells. Although the tropism of *C. pneumoniae in vivo* appears to alveolar macrophages, the isolation of live *C. pneumoniae* organisms and also their DNA from the excised coronary artery of atheroma patients provides evidence for transmission of *C. pneumoniae* to atherosclerotic foci (Maass et al., 1998). The route of transmission of *C. pneumoniae* from the lung to atherosclerotic foci is not clear, but *in vivo* studies have suggested that *C. pneumoniae*-infected peripheral blood monocytes may serve as a vehicle for transmission through circulation (Moazed et al., 1997; Redecke et al., 1998). The lifecycle of *C. pneumoniae* in macrophages lasts at least for 3 days *in vitro* and 7 days *in vivo*, leading to long and sustained inflammatory response as opposed to acute, short response elicited by extracellular pathogens (Hogan et al., 2004). During the infection period, the infected monocytes/macrophages secrete copious pro-inflammatory cytokines, ROS and matrix-degrading proteases, degrade collagen, adhere to and transmigrate through endothelium, and promote smooth muscle cell migration (Lindholt et al., 1999; Movahed, 1999). These *in vitro* studies have clearly highlighted the role, and partially elucidated the possible mechanisms by which *C. pneumoniae* infection may promote atherosclerosis. In addition, some animal model studies highlighted the importance of active chlamydial infection to exacerbate atherosclerosis, though a thorough study on mechanisms is not very well studied (Sharma et al., 2004).

Heretofore, our understanding of *C. pneumoniae* infection and inflammation sequelae is, almost exclusively, based on suspension cultures, though these assays may not capture the complex environment of the vascular wall in three dimensions. Since atherosclerosis is an outcome of chronic inflammation of the arterial wall, decoding the role of *C. pneumoniae* infection inside the intima is a critical step in understanding the spatio-temporal evolution of the disease. It is well-established that the 3D matrix environment can modulate cellular responses not only due to modified concentration gradients or presentation of ligands (i.e., biochemical stimuli) but also due to physical confinement and ensuing mechanical stress (i.e., biophysical stimuli) (Matthews et al., 2006; Gasiorowski et al., 2013; Hoon et al., 2016). We hypothesize that the biomechanical and biochemical effects of *C. pneumoniae*-infected macrophages on the vessel wall matrix hasten atherosclerosis. To test this hypothesis, we used a 3D microphysiological model of the vascular wall, and evaluated the cross-talk between biochemical and biophysical stimuli are particularly relevant in atherosclerosis as both these factors have been shown to influence the development of the disease: pro-inflammatory cytokines and ROS promote recruitment of macrophages, LDL oxidation and foam cell formation (biochemical signaling); and the matrix-degrading proteases alter the mechanical properties of the extracellular matrix, which in turn, trigger significant changes in cellular function (biophysical signaling) (Agabiti-Rosei and Muiesan, 2007; Zhao, 2009; Van Goethem et al., 2010; Chiu and Chien, 2011). To understand the importance of long infection cycle to chronic inflammation, we compared the aforementioned cross-talk when macrophages are infected with chlamydial antigens and uninfected macrophages.

## Materials and Methods

### Cells

Human blood samples, without any donor identifiers, were obtained after their informed consent, and the buffy coat was isolated by the South Texas Blood and Tissue Bank (STBT), San Antonio, TX, USA. Buffy coat was procured from STBT. All the methodologies and procedures were approved by Institutional Review Board (IRB), The University of Texas at San Antonio, San Antonio, TX, USA (protocol #12-227), and the experiments were conducted in accordance with these appropriate guidelines. PBMCs were isolated following the protocol detailed in Evani and Ramasubramanian (2016). Primary human umbilical vein endothelial cells (HUVECs) were obtained from Life Line Cell Technologies and cultured according the manufacturer’s instructions, stored at -80°C within three passages (eight doublings). The cells were thawed, used within one passage for the experiments.

### Bacteria

*Chlamydia pneumoniae* TW183 EB was obtained from the University of Washington (Seattle, WA, USA), aliquoted and stored at -80°C. *C. pneumoniae* stock titer was determined following established protocols using TT401 antibody (Campbell and Kuo, 2009). For experiments to study the effect of chlamydial antigens, the bacteria were rendered ineffective by heat treatment at 95°C for 30 min.

### Chlamydial Infection of Monocytes

Human monocytes were infected with *C. pneumoniae* EB at a Multiplicity of Infection (MOI) 1 as described in detail elsewhere (Evani and Ramasubramanian, 2016). A similar procedure was followed for infection with heat-killed bacteria. Monocytes that were treated with buffer (uninfected or mock) were used as controls.

### Preparation of Collagen Matrix and Encapsulation of Monocytes/Macrophages

Collagen gels of concentration 2 mg/ml with 3 million/ml monocytes/macrophages were prepared using rat tail collagen type l (Corning, USA) according to manufacturer’s protocol. Briefly, appropriate amounts of high concentration rat tail collagen solution, recommended quantity of neutralizing 1N NaOH and 100 μl of 10x dPBS were mixed on ice. Human monocytes from 4 h post infection were resuspended in complete media at a concentration of 3 × 10^7^ cells/ml (untreated, HK *Chlamydia* and Cpn infected). Hundred microliters of this cell suspension was added to the neutralized collagen solution. Sterile water was then added to the collagen solution to make up the volume of final solution to 1 ml and supplemented with gentamycin (1 μg/ml). Appropriate volumes of the collagen solution on ice was quickly loaded in culture dishes/well plates/glass slides and allowed to gel for 20 min at 37°C. Complete media was then added to gels to immerse and further incubated for up to 12 days.

### Dextran and LDL Permeability

Human umbilical vein endothelial cells were grown to confluence for a minimum of 3 days in the top well of a Transwell [Polyethylene terephthalate (PET) membranes, 6.5-mm diameter, 3.0 μm pore size; Corning, Falcon]. The confluency of the monolayer was verified by staining one of the inserts with Phalloidin-Alexa fluor 488 and DAPI and visualized by fluorescence microscope. Confluent HUVEC inserts were placed in companion plates that contain 200 μl collagen gels containing human monocytes (uninfected, heat killed, or infected with *C. pneumoniae*) supplemented with 400 μl of media (lower chamber). After 24 h, inserts were removed and placed in fresh 24-well companion plate containing 600 μl fresh lifeline vascular basal media (lower chamber) and 100 μl of 1 mg/ml Rhodamine B isothiocyanate–dextran in top chamber (insert). After 1 h incubation at 37°C, 100 μl aliquots were taken from the lower chamber and added to a black 96-well plate. Endothelial permeability was measured by estimating the fluorescence at ex/em of 528/600 nm using a fluorescence plate reader. TNF-α stimulation was used as a positive control and collagen gels without cells as negative control. For LDL permeability assays, dextran was replaced with 100 μl of BODIPY FL-LDL (Life Technologies), and the procedure described above was followed. LDL permeability was measured at ex/em of 485/528 nm using Synergy BioTek plate reader.

### Endothelial Integrity from VE-Cadherin

Human umbilical vein endothelial cells on PET membrane transwell inserts from the above experiment were fixed with 4% formaldehyde, and incubated with mouse antihuman vascular endothelial (VE)–cadherin antibody (1:100; Abcam), followed by incubation with secondary goat anti-mouse Alexa 488–conjugated antibody and Alexa 633–conjugated phalloidin (1:100) and DAPI for nucleus (1:100; Molecular Probes). After incubation with each antibody, the membranes were washed three times for 10 min with BD Permwash. Finally, membranes were dipped once in water and mounted with Fluorosave (Calbiochem) on microscopic glass slides. The membranes were then visualized under a Zeiss-510 confocal microscope at a magnification of 100×.

### Cell Viability

Five microliters of collagen gels were spotted in a black 96 well plate, supplemented with 50 μl media and incubated for up to 1 week. The matrices were then treated with 50 μl PBS containing Calcein-AM for 30 min at RT. The wells were gently/carefully washed with media, supplemented with 50 μl of PBS, and fluorescence readings of the spots (area scan) were taken at ex/em of 488/530 nm using BioTek plate reader. Fluorescence readings from fresh gels with various concentrations of monocytes were used to generate standard curve, from which the viability was determined. The gels were immediately fixed and mounted on a cover glass for 3D confocal imaging.

### Cytokine and Chemokine Levels

After incubation of up to 1 week, supernatant from collagen matrices were collected, and assayed using ELISA kits for detection of cytokines and chemokines IL-1β, IL-8, MCP-1, and TNF-α following manufacturer’s protocol (BD biosciences).

### ROS and SOD

Five microliters of collagen gels were spotted in a black 96 well plate, supplemented with 50 μl media and incubated for up to 1 week. Intracellular ROS was stained using Image-IT green and mitochondrial SOD was stained with Mito-SOX red assay kits (Life technologies, USA), along with staining for nucleus using DAPI according to manufacturer’s instructions. Post staining, the gels were fixed with 4% formaldehyde and mounted on a cover glass for 3D confocal imaging. 3D confocal images were taken at ex/em set at 380/405, and 495/529 nm and 510/580 nm for gels stained with DAPI, Image-IT green and Mito-SOX red assay kit, respectively. Fluorescence intensity within the cells was measured from images using Image Pro analyzer, which was expressed in Fluorescence Intensity Unit (FIU).

### MMP Release

Matrix metalloproteinases secreted by macrophages into the supernatant was measured by a colorimetric assay following the manufacturer’s instructions (Anaspec fluorimetric MMP assay kit). The MMPs analyzed by the kit were MMP-1, 2, 3, 7, 8, 9, 12, 13, and 14. Enzyme activity of the supernatants collected was measured at 37°C. Activity units in the plateau region of the enzyme activity were chosen as a representation of the MMP release/activity.

### Collagen Degradation

Five microliters of gels were placed in black 96 well plates, and incubated in media for up to 7 days. The matrices were then fixed with 2% formaldehyde, and stained with 10 μg/ml of anti C-terminal collagen antibody overnight (AdipoGen Life sciences). The gels were washed three times for 10 min each time using PBS-T and then stained with Alexa fluor 488 secondary anti rabbit-antibody (Abcam) for 2 h, along with Alexa fluor 633-phalloidin and DAPI. The gels were washed three times for 10 min each time using PBS-T and mounted on a cover glass with a drop of FluorSave. Z-stack images were recorded at 100× magnification at ex/em set at 380/405, and 488/520 nm and 510/580 nm for nucleus, collagen nicks and cytoskeletal actin, respectively, using confocal microscopy.

### Cell Morphology and Matrix Microstructure by Image Analysis

To quantify the cell spreading in 3D, gels with phalloidin/calcein AM stain were used to obtain at least five Z-stack images with a stack interval of 1.8 μm with a total *Z*-height of at least 60 μm. Using Image Pro analyzer, the 3D stack was compressed to a 2D image, and processed for estimating area occupied by single cells and by cluster of cells: for single cells normalized actin intensity per cell was used; and for cell clusters, calcein intensity was used. A group of cells was considered to be a cluster if >5 cells (or 500 μm^2^) were present as a continuum. To quantify the changes in the microstructure of gels doped with fluorescence collagen (Fl-collagen), at least five Z-stack images with a stack interval of 1.8 μm were obtained for in phase collagen fibers with a total Z-height of at least 60 μm. Using Image Pro analyzer/Image J, the 3D stack was compressed to a 2D image, and processed to obtain the distribution of fiber density, orientation, and alignment. To estimate fiber density, the 2D stacked image was converted to gray scale (0–255), and the intensity distribution was obtained. The lowest gray scale (<28) was expeditiously chosen to represent absence of any prominent fibers. Fiber orientation was estimated for a 2D stacked gray image using Orientation J plugin with a defined set of parameters in Image J software, and was defined as the fiber angle between 0° and 90°. The frequency distribution of the angles was used as a measure of skewness in fiber alignment. To estimate fiber length, Skeleton analysis plug in in Image J software was used. To estimate fiber thickness, the 2D stacked gray image was converted to gray scale, and the grayscale threshold was set to 75 units so that only bundled fibers (i.e., filaments) were identified. Filament properties were estimated by calculating filament thickness and area occupied by bundled filaments using Image J software.

### Viscoelastic Properties

The viscoelastic properties of collagen matrices (*n* = 5) were measured using Physica MCR 300 rheometer (Anton-Paar, Ashland, VA, USA). The samples were prepared with dimensions to the 15 mm circular geometry of the parallel plate spindle being used. Briefly, human monocytes (control, uninfected, HK-infected, and live-infected) were embedded in 200 μl (three spots/dish) collagen gels and placed in 35 mm petri dishes with 12 mm hydrophobic borders (drawn with a hydrophobic pen and kept under UV light over night before making gels). After gelation at 37°C, the gels were detached from the surface, supplemented with media and the dishes were placed in a 5% CO_2_ incubator at 37°C and for up to 7 days. After 3 or 7 days of incubation, the gels were analyzed by rheometry under humidified conditions. To avoid any slippage during the experiment, a rough sand paper cut to the shape of the spindle was attached to the plates. Oscillatory amplitude testing over 0.1 to 100% strain of the gels was performed to determine the linear viscoelastic range of the storage modulus (*G’*). The elastic and loss moduli were measured from the frequency sweep ranging between 1 and 100 rad/s at 0.5% strain. The yield point was obtained from the amplitude sweep ranging between 0.1 and 1000% strain. In another experiment, the gels from 1 week post infection were placed in IBIDI sticky slide and 1x PBS was infused for 5 min at 50 dyn/cm^2^ of wall shear stress. The samples were then fixed in neutral buffer formalin and processed for scanning electron microscopy analysis by dehydrating the samples with ethanol and coated with gold. The samples were then analyzed for collagen tear/rupture by SEM at a magnification of 5000×.

### Statistical Analysis

Statistical analysis was performed using Graph Pad Prism 6 (Groningen, The Netherlands). Each experiment was performed in triplicates and was conducted twice, and a Student’s *t*-test or a two way ANOVA (followed by Bonferroni correction/Tukey’s *post hoc* analysis) was used to estimate whether a significant differences between the treatments existed The differences were considered significant if *P*< 0.05 (*t*-test/ANOVA).

## Results

### Infected Cells in 3D Environment

**FIGURE 1:**
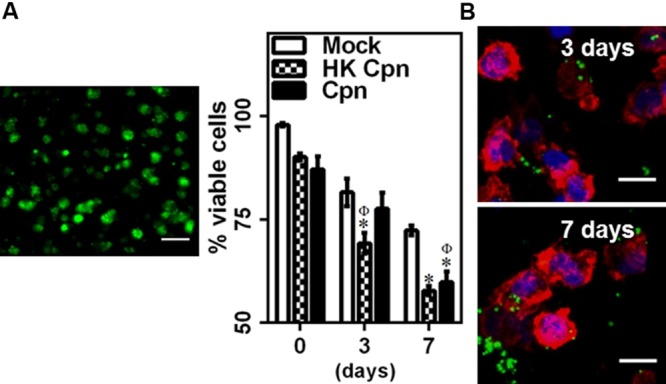
***Chlamydia pneumoniae* infection of macrophages in 3D collagen matrices.** Monocytes were infected with PBS (mock) or heat-killed Cpn (HK Cpn) or Chlamydial EB of MOI 1 (Cpn) and embedded in 2 mg/ml collagen. Post gelation, cells were supplemented with media and incubated for up to 1 week. **(A)** Gels were stained with Calcein AM (green) and viability was estimated. A representative 2D projection confocal image of viable cells in the gel from 3D stack is shown (Scale bar: 20 μm). The results are mean ± SEM of three experiments performed in triplicate. The ^∗^ and Φ symbols denote statistically significant change in viability respective groups when compared to Mock from same day and within the group relative to previous time point, respectively (*P*< 0.05). **(B)** Macrophages were stained with anti-Cpn antibody (Green), AF 633 phalloidin (Red), and DAPI (Blue). Representative confocal images of infected cells at 3 and 7 days are shown (Scale bar: 10 μm).

To understand the effect of matrix environment on infected macrophages, cells infected with PBS (Mock), heat-killed (HK Cpn) and live *C. pneumoniae* were encapsulated in 3D collagen matrices, and viability and infectivity were analyzed for up to 1 week. The viability of macrophages decreased gradually during the course of 7 days, although even at the end of 1 week more than 60% of the infected cells were viable suggesting that infected macrophages are capable of surviving in the matrix for substantially long periods of time (>40% cells were viable at the end of 12 days: data not shown). Monocytes infected with heat-killed chlamydial EBs show similar viability pattern to those of monocytes infected with live organisms with decrease in viability as early as 3 days (**Figure 1A**). Using confocal microscopy, we observed that at least 80% of the cells were infected and, consistent with our previous findings, the size of the inclusions ranged from very small to large inclusions (3–30 μm), and the cells contained a variable number of inclusions (**Figure 1B**) (Cheeniyil et al., 2015). Interestingly, *C. pneumoniae* can be seen outside cells in the matrices at the end of 1 week possibly due to the release of *C. pneumoniae* inclusions, which correlates with the decrease in viability of infected cells in 7 days. As expected, cells infected with HK *C. pneumoniae* did not form any inclusions.

### Inflammatory Response of Infected Macrophages in 3D Environment

**FIGURE 2:**
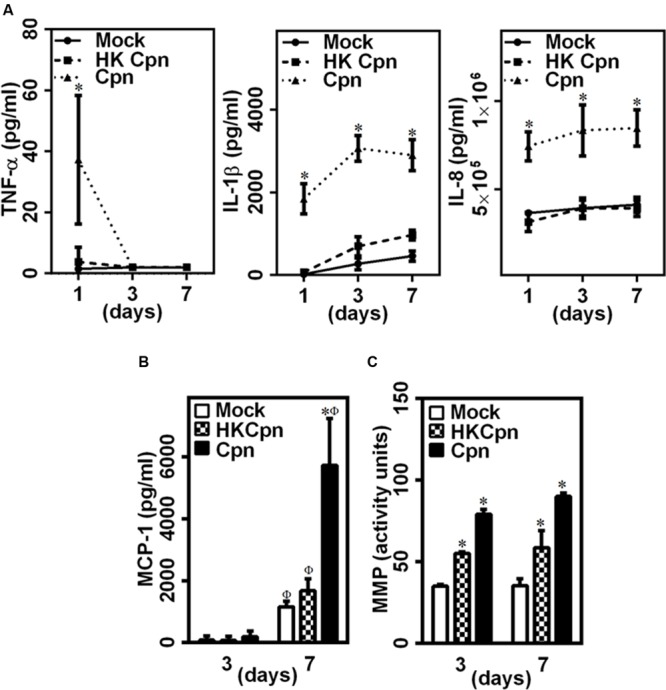
**Pro-inflammatory environment in 3D collagen matrices with infected macrophages. (A)** The supernatants were collected from the 3D gels on days 1, 3, and 7, and estimated for the levels of TNF-α, IL-1β, and IL-8 by ELISA. The results are plotted as quantity of secreted protein as obtained from standard curve. **(B)** MCP-1 secretion as measured from supernatants at day 3 and 7 post infection by ELISA. **(C)** MMP activity assayed from supernatants at day 3 and 7, using Pan-MMP activity assay kit. The results are mean ± SD of one representative experiment performed in triplicate, and the experiments were repeated three times. The ^∗^ and Φ symbols denote statistically significant change in viability respective groups when compared to Mock from the same day and within the group relative to previous time point, respectively (*P*< 0.05).

Upon chlamydial infection, leukocytes in suspension cultures (Netea et al., 2000; Bas et al., 2008) secrete pro-inflammatory cytokines and chemokines, MMP, and reactive oxygen species (ROS), all of which contribute to development and progression of atherosclerosis. To examine the effect of *C. pneumoniae* infection in cells trapped in an extracellular matrix, we quantified the secretion of key pro-inflammatory cytokines and chemokines from infected macrophages encapsulated in 3D collagen gels. We observed that the infected cells secrete small quantities of TNF-α within the first day, which declined soon after (**Figure 2A**). TNF-α secretion was undetectable in uninfected and HK-infected controls. In contrast, cells infected with live *C. pneumoniae* secreted large quantities of IL-1β and IL-8 for 7 days, unlike cells infected with HK *C. pneumoniae* or uninfected cells. We also observed that cells infected with live *C. pneumoniae* produced significantly higher levels (threefold) of MCP-1 by 7 days compared to cells infected with HK *C. pneumoniae* or uninfected controls (**Figure 2B**). Cells infected with either live or HK organisms produced significantly more MMPs compared to uninfected cells. Interestingly, HK *C. pneumoniae* organisms triggered MMP production compared to uninfected controls (**Figure 2C**). *C. pneumoniae* infection triggers the secretion of, amongst others, MMP-1 and -8 which degrade collagen and play an important role in atherosclerosis (Mäntylä et al., 2004; Rupp et al., 2004; Kern et al., 2009). This data reveals both live *C. pneumoniae* and chlamydial antigen trigger a proinflammatory response in encapsulated macrophages, with live infection eliciting a higher and lasting response.

### Endothelial Permeability during Active Chlamydial Infection in Macrophages

**FIGURE 3:**
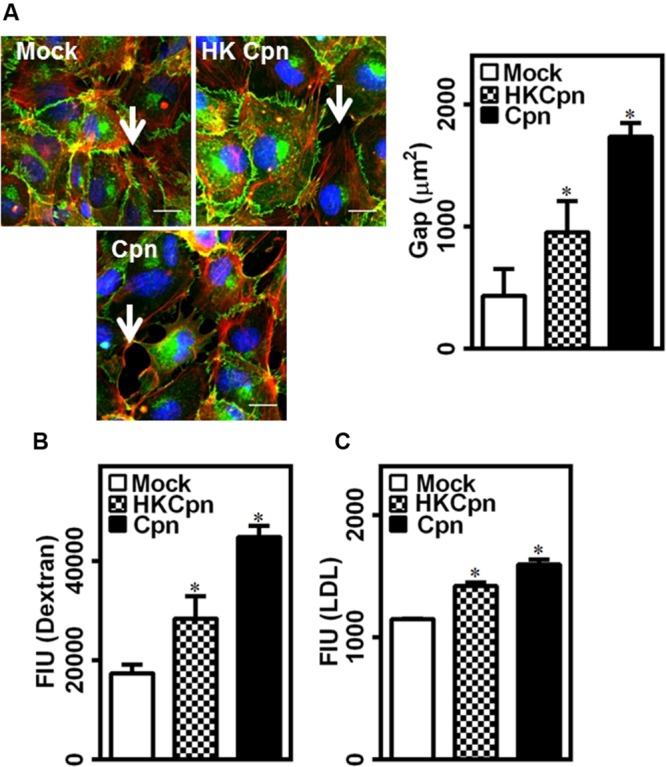
**Endothelial junction dysfunction during *C. pneumoniae* infection in matrices.** Monocytes were infected with PBS (mock) or heat-killed Cpn (HK Cpn) or chlamydial EB of MOI 1 (Cpn) for 4 h and then embedded in 2 mg/ml collagen at a concentration of 3 million cells/ml of gel and then incubated for 24 h in a 24 well plate with *trans*-well containing confluent endothelial cells. **(A)** After incubation, the endothelial cells were stained with antibodies against VE-cadherin (green), phalloidin (red) and DAPI (blue), and visualized using Zeiss-510 confocal microscope. Representative images (Scale bar: 20 μm) with gaps (white arrow) between the cells were analyzed for gap area using Image-Pro analyzer, and results were plotted as average of three images. **(B,C)** In another experiment, post incubation, media from upper side of *trans*-well was replaced with fluorescent dextran **(B)** or BODIPY-LDL **(C)**, and the plate was incubated for 1 h and supernatants were collected from bottom well. Fluorescent intensity from supernatant was measured using plate reader as an estimate of permeability. The ^∗^ denote statistically significant change in the parameters between different groups, as calculated using Graph-Pad Prism (*P* < 0.05, ANOVA).

The breach of endothelial barrier is one of the early hallmarks of atherosclerosis. We hypothesized that the high levels of IL-1β and IL-8 secreted by infected macrophages will trigger endothelial dysfunction. To this end, we incubated an intact and confluent endothelial layer over the collagen matrix containing uninfected/infected cells for 24 h, and analyzed the barrier integrity based on the distribution of junctional protein VE-cadherin and permeability of dextran (**Figure 3A**). While the uninfected macrophages had a small effect (as indicated by arrows), macrophages infected with either HK or live chlamydial infection compromised the endothelial barrier significantly, with live infection disintegrating the endothelial junction substantially. We quantified the total gap between endothelial cells from the images stained with VE-cadherin. We observed that macrophages infected with live chlamydial infection in the sub-endothelium created threefold more gaps in endothelium than those with HK bacteria or mock infected macrophages. These leaky junctions disrupt the barrier permeability and might increase the transendothelial transport molecules, which otherwise cannot pass the junction via para-cellular route when endothelial junction is intact. We estimated the permeability of fluorescent dextran to correlate permeability with barrier disruption (**Figure 3B**). We observed that permeability of dextran increased 2.5-fold in the presence of active infection in sub-endothelium when compared to HK and mock infected controls. This disruption might facilitate para-cellular LDL movement into the arterial wall along with permeation of LDL by *trans*-cellular route by activated endothelium, as seen from a significant increase in LDL transport across the endothelial barrier due to infection (**Figure 3C**). Chlamydial antigens, though did not produce substantial IL-1 and IL-8, were enough to disrupt the endothelial barrier and allow transendothelial movement of LDL (**Figures 3B,C**). Of note, macrophages encapsulated in 3D matrices secreted threefold higher IL-8 compared to 2D cultures, which translated to a twofold increase in the transendothelial permeability of dextran (Supplementary Figures S1A,B). Although limited, our data indicates that the 3D cultures may elicit a higher inflammatory response than 2D cultures, thus underscoring the importance of relevant *in vitro* tissue models of infection. Further, basal activation of the endothelium is more effective in increasing the permeability when compared to apical activation, suggesting a major role of lodged macrophages in 3D on endothelial damage in atherosclerosis (Supplementary Figure S1C).

### LDL Uptake by *C. pneumoniae*-Infected Macrophages

**FIGURE 4:**
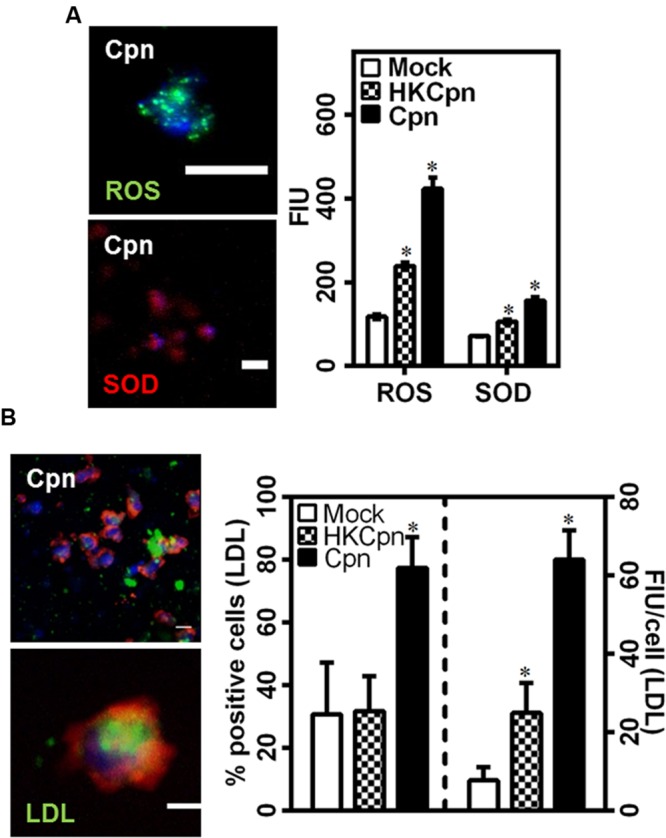
**Low density lipoprotein uptake of *C. pneumoniae* infected macrophages in collagen matrices. (A)** The gels after 7 days of infection were stained for ROS (green) and SOD (red) as per manufacturer’s instructions. A representative image of Cpn infected macrophages in gels and the graph showing quantification of fluorescence intensity from the gels. Scale bar: 10 μm. **(B)** Monocytes were infected with mock PBS or HK/Live Chlamydial EB (MOI 1) for 4 h and embedded in 2 mg/ml of collagen with 10 mg/dl of BODIPY-LDL (green) and incubated for up to 1 week. Macrophages were fixed and stained for actin (Red), and nucleus (Blue). A representative confocal image of macrophages 7 days after infection (Scale bar: 10 μm), and quantification of LDL uptake by macrophages. The results are mean ± SD of one representative experiment performed in triplicate, and the experiments were repeated three times. The ^∗^ denote statistically significant change in the parameters between different groups, as calculated using Graph-Pad Prism (*P* < 0.05, ANOVA).

Low density lipoprotein is oxidized to oxLDL, which is taken up by macrophages to form foam cells, a crucial part of plaque. We found that cells infected with live *C. pneumoniae* in the matrix produced significantly higher levels of ROS and SOD when compared to cells infected with HK organisms, which in turn was higher than uninfected controls (**Figure 4A**). We hypothesized that the matrix containing infected cells rich in ROS and SOD will promote foam cell formation. To estimate uptake of LDL by macrophages, we exposed encapsulated macrophages to LDL, and analyzed the uptake 7 days after infection (**Figure 4B**). Analysis of accumulated LDL in macrophages shows that higher percentage of infected cells loaded with LDL when compared to HK and mock infected macrophages. Further, macrophages infected with live EBs were more effective in taking up LDL as evident from LDL load per cell (∼2.5-fold) in infected macrophages than uninfected macrophages or macrophages infected with chlamydial antigens. Again, infection of macrophages by chlamydial antigens still resulted higher LDL accumulation than uninfected controls, possibly due macrophage activation and hence oxidation of LDL (**Figure 4B**). These observations support the premise of accelerated atherosclerotic progression due to live infection or bacterial antigens in the intima.

### Macrophage Migration

**FIGURE 5:**
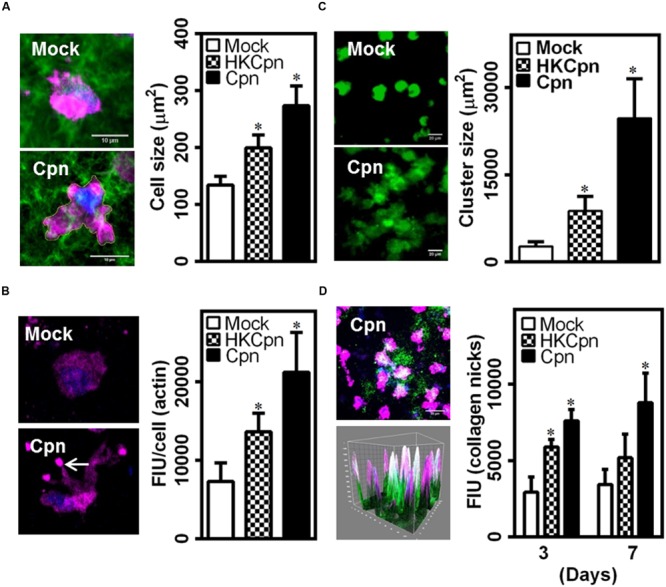
***Chlamydia pneumoniae* infection alters macrophage phenotype in collagen matrices.** After infection for 4 h, monocytes were embedded in collagen and incubated for up to 1 week. **(A,B)** Macrophages in 3D gels were fixed and stained for actin (Red), and DAPI (Blue). **(A)** Representative confocal image of infected cells at 7 days post infection (Scale bar: 10 μm), with graph showing the area of spread cells. **(B)** Visualization of quantification of cellular actin. **(C)** Macrophages were stained with Calceine AM and Z-stack confocal images of gels at 7 days after infection are shown (Scale bar: 20 μm) with graph showing area occupied by cell clusters. **(D)** Gels were stained for nicks in collagen (green), actin (pink), and DAPI (blue), and visualized by confocal microscopy. A representative 3D image (Scale bar: 20 μm), corresponding 3D surface plot showing nicked–collagen (green), and its localization with cells (pink/blue), and quantification of intensity of collagen breaks as obtained from image analysis using Image Pro Analyzer. The results are mean ± SD of three experiments performed in triplicates and ^∗^ denote statistically significant change in the parameters between different groups, as calculated using Graph-Pad Prism (*P* < 0.05, ANOVA).

Since, we observed substantially high levels of MCP-1 released by *C. pneumoniae*-infected macrophages (**Figure 2B**), we analyzed the migration of infected cells in the matrix by quantifying spreading and cytoskeletal changes in individual macrophages, and the density of macrophages within the collagen matrix. After 7 days, live-infected and HK-infected cells spread more when compared to uninfected cells, which retained their spherical shape and spread less (**Figure 5A**). In uninfected macrophages, actin predominantly concentrated around the nucleus but in infected macrophages, actin was visibly concentrated in the filopodial segments and was higher, which are characteristics of motile cells (**Figure 5B**). We also observed that infected macrophages form clusters, which are densely packed cells in certain areas of the 3D collagen matrix, thus supporting the cause of higher actin polymerization, spreading, and movement of infected cells (**Figure 5C**). This clustering may also be a result of aggregation due to the upregulation/activation of adhesion receptors, including integrin, and ICAM-1 in infected cells (Evani et al., 2011, 2013). Although macrophages infected with chlamydial antigen did not show clustering at the end of 1 week; at the end of 2 weeks, they also exhibited areas with significantly high cell density (data not shown). As shown in **Figure 2C**, *C. pneumoniae* infection elicits the production of MMPs, which is a key in the degradation of collagen matrix and subsequent remodeling. To understand the effect of MMP produced during *C. pneumoniae* infection on the microenvironment, we quantified the extent of degradation from the binding of fluorescently labeled antibody to c-terminal of collagen strands (**Figure 5D**). We observed higher collagen degradation around the infected cells suggesting a higher concentration of secreted MMPs in the neighborhood immediate to the cells after both 3 and 7 days.

### Remodeling of Intimal Matrix

**FIGURE 6:**
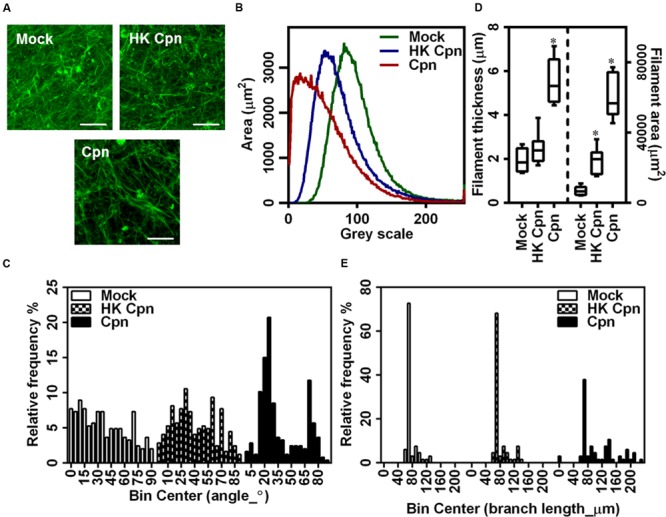
**Macrophages with *C. pneumoniae* infection alter collagen micro environment.** After infection for 4 h, monocytes were encapsulated in collagen matrix doped with 10% FL–collagen and incubated for up to 1 week. **(A)** Representative 3D images of fluorescent collagen fibers from confocal microscopy (Scale bar: 20 μm), with quantification of: fluorescent collagen fiber gray scale area **(B)**; filament thickness **(C)**; filament length **(D)**; and fiber angle **(E)** from image analysis using Image J analyzer. The results are mean ± SD of one experiment performed in triplicate, and the experiment was repeated at least three times.

To understand the role of infection on the matrix microstructure, we analyzed the morphological properties of fluorescently stained individual collagen fibers from CLSM images (Supplementary Figure S2). Visual observation suggested that infection resulted in less uniform and more regular alignment of the fibers compared to uninfected or no cell controls (**Figure 6A**). To quantify the uniformity of fibers, we quantified the intensity distribution in individual images, and a representative distribution is shown in **Figure 6B**, which shows that the infection results in an increase in areas that lack collagen fibers. The orientation of fibers was calculated from the angles that the fibers make to the *X*–*Y* axes in each of the quadrants. These angles vary between 0° and 90°, and reveal alignment of fibers (**Figure 6C**). Matrix containing uninfected cells showed uniform alignment, which becomes non-uniform following an infection. Matrix with infected cells showed a high percentage of fibers at 15° to 25° and 70° to 80° suggesting that infected cells impose fiber orientation. Fresh collagen production by activated macrophages and macrophage migration might distort and bundle up fibers as previously shown in studies on SMCs. Finally, we estimated the length of the fibers, thickness of individual clusters of fibers, and the area occupied by these clusters (**Figure 6D**). These estimations show that while the matrix containing uninfected cells are predominantly made of fibers ∼60 μm in diameter, infected cells bundle the collagen fibers laterally to form thicker filaments, and such thicker filaments dominate the distribution. Matrix containing cells infected with HK-*C. pneumoniae* showed lower but similar thickness throughout suggesting less/no bundling of collagen fibers (**Figures 6D,E**). Together, our analysis demonstrates a profound effect of infection on collagen architecture of the 3D gels.

### Matrix Mechanical Changes during Active Chlamydial Infection

**FIGURE 7:**
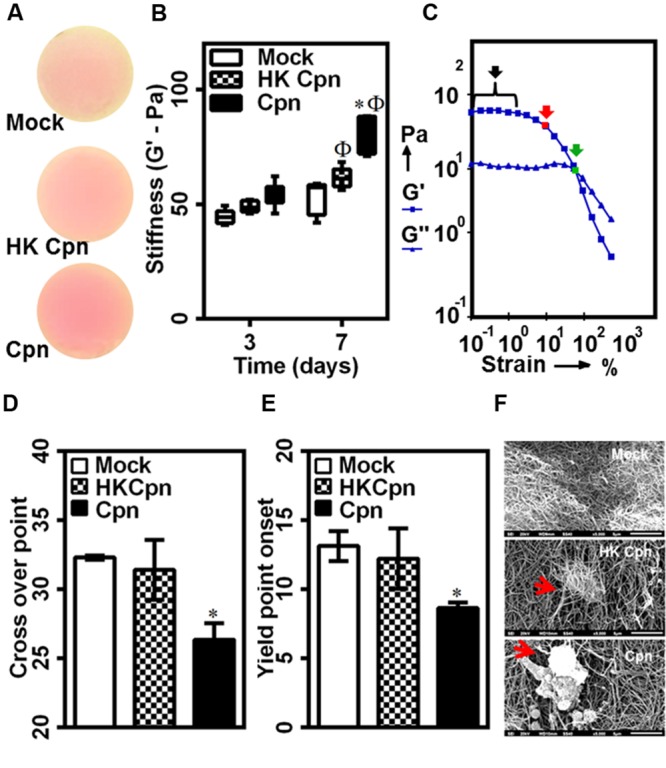
**Matrix mechanical changes due to live *C. pneumoniae* infection in intima.** Monocytes were infected with mock PBS or HK/Live Chlamydial EB (MOI 1) for 4 h. Post infection, cells were embedded in 200 μl of 2 mg/ml collagen, and supplemented with media and incubated for up to 1 week. **(A)** Visual inspection of gel integrity after 1 week of incubation before rheological analysis; **(B–F)** stiffness and strength of gels was estimated by dynamic shear rheometer. **(B)** Stiffness; **(C)** Representative amplitude sweep with linear viscoelastic region (black arrow), yield point onset (red arrow), cross over point (green arrow), **(D)** Dross-over point, and **(E)** Yield point onset. The results are mean ± SD of five experiments performed in triplicates. The ^∗^ and Φ denote statistically significant change in viability respective groups when compared to Mock from same day and within the group relative to previous time point, respectively, as calculated using Graph-Pad Prism (*P* < 0.05, ANOVA) **(F)** In another experiment, the gels were prone to a shear stress of 50 dyn/cm^2^ in a micro-fluidic chamber using syringe pump. The gels were fixed and analyzed by scanning electron microscopy with representative images (Scale bar: 5 μm) from different conditions.

Having established that infected macrophages has a profound effect on matrix architecture, we investigated the role of infection on the mechanical properties of the collagen matrix. To this end, we used dynamic mechanical analysis to estimate the viscoelastic properties of collagen gels. Prior to rheological analysis, we confirmed that uninfected/infected macrophages did not induce any morphological changes such as shrinkage or damage to the gels over 7 days (**Figure 7A**). To measure the elastic (*G’*) and loss moduli (*G”*), we subjected the gels to a steady strain rate of 0.5%, which falls in the linear viscoelastic limit of collagen gels. Consistent with previous reports, the elastic modulus or gel stiffness of collagen gels was estimated to be 50 Pa, which did not change due to macrophage encapsulation (Supplementary Figure S3). The concentration of naïve monocytes was adjusted so that the mechanical properties are not dependent on the cell concentration. After 3 days of infection, we did not observe any significant difference in gel stiffness due to infection. However, at 7 days post infection, the stiffness of gels containing macrophages infected with live *C. pneumoniae* EB increased to ∼80 Pa, which is 1.5-fold higher when compared to gels containing macrophages infected with chlamydial antigen or uninfected controls. After 7 days, the stiffness of the gels containing macrophages infected with chlamydial antigen gels increased compared to 3 days uninfected controls, whereas the stiffness of the gels with uninfected macrophages was unaltered (**Figure 7B**).

Next, we measured the yield strength of the gels as the point of irreversible deformation when the gels were subjected to increasing strain, i.e., during amplitude sweep. From the response, we obtained the yield point as the strain rate at which *G’* starts to decrease, and crossover point as the strain rate at which a loss in gel integrity was observed (**Figure 7C**). Surprisingly, we observed that collagen gels containing live infection showed an early loss of mechanical strength as evident from yield point onset at ∼7% strain (**Figure 7D**) and cross-over point (**Figure 7E**) at lower strain percentage (∼26%) when compared to other conditions. To further confirm this observation, we exposed gels from 7 days post infection to high levels of wall shear stress (∼50 dyn/cm^2^), as may be encountered at plaque site *in vivo*, by infusing buffer over gels formed in a microfluidic channel. The samples were analyzed for collagen tear/rupture by SEM. Qualitative observation of the images revealed tear in collagen gels thus exposing live *C. pneumoniae* infected macrophages due to shear-induced rupture, and particularly in the immediate vicinity of the cells. This was not as pronounced in controls (**Figure 7F**) highlighting the impact of micro-environmental changes due to live chlamydial infection.

## Discussion

Infectious agents are increasingly implicated in either causation or hastened atherosclerotic progression (Campbell and Rosenfeld, 2015). In this study, using *in vitro* model of intima, we demonstrated that inflammation due to *C. pneumoniae* infection in macrophages yields an atherogenic phenotype including endothelial dysfunction, matrix remodeling, and foam cell formation. To our knowledge, this is the first study that has addressed both the biomechanical and biochemical effects of the inflammatory response of infected macrophages in a 3D microenvironment. Heretofore, our understanding of *C. pneumoniae* infection and inflammation sequelae are, to a great extent, based on non-physiological 2D monolayer or suspension models focusing only on secreted biochemical factors, though it has been rightfully argued that these assays may not be relevant to the complex environment of the vasculature (Ingber, 2003). More evidence is accumulating that the 3D matrix environment can modulate cellular responses not only due to physical confinement and presentation of ligands but also by mechanotransduction (McWhorter et al., 2015). Recently, there has been some efforts toward the development of *in vitro* 3D tissue mimics for a faithful representation of host–pathogen interactions (Barrila et al., 2010; Benam et al., 2015). Thus, by using a 3D culture system, we have more closely mimicked the *in vivo* environment by replicating cell-matrix interactions, cell polarity, spatial and temporal distribution of signaling molecules, and remodeling of the ECM.

Atherosclerosis is an ongoing inflammatory response (Libby et al., 2002). Infectious agents may furnish inflammatory stimuli that accentuate atherogenesis, and intravascular infections may be more effective than extravascular infections because of their localized action on atherosclerotic foci. *C. pneumoniae* is an intracellular pathogen, and *in vitro* can reside in macrophages for up to 3 days. Our data shows that infected macrophages in collagen matrix is viable for at least 7 days (and up to 12 days, the maximum period we observed), and creates and sustains an inflammatory microenvironment that contributes to different stages of atherosclerosis including endothelial dysfunction, foam cell formation, and alters matrix stiffness. With sustained inflammation being a key factor in mediating atherosclerotic pathology, and *C. pneumoniae* being a ubiquitous respiratory pathogen with common reinfections, our data illustrates the unique contribution of *C. pneumoniae* infection to atherosclerosis by modifying both the biochemical and biophysical properties in the milieu.

The infected macrophages lodged in the subendothelial matrix releases a number of soluble factors which gradually transforms tissue homeostasis. First, the basal activation of endothelial cells due to *C. pneumoniae* infection in the subendothelial matrix can be particularly potent in triggering the entry of macromolecules such as LDL and circulating monocytes into the intima (Almenar-Queralt et al., 1995; Sima et al., 2009; Shapiro and Aird, 2011). Second, sustained oxidative stress is key to the oxidation of LDL, which contributes to atherogenesis by the recruitment and retention of macrophages, ready internalization by macrophages to form foam cells, and the loss of endothelial integrity (Stocker and Keaney, 2004). Third, the migration of macrophages toward each other to form focused clusters promote the transformation to lipid-laden foam cells, the prototypical cells in the atherosclerotic plaque. This migration also suggests that the chemokines released from infected macrophages may efficiently trap uninfected macrophages leading to defective efferocytosis.

The biophysical interaction of lodged macrophages with the extracellular matrix is another key mechanism in the development of atherosclerosis. The extracellular matrix is extensively remodeled as collagen crosslinks are degraded by MMPs secreted by lodged macrophages, and are partially rescued by the synthesis of new collagen by smooth muscle cells (Paolillo et al., 2012). However, the pro-inflammatory cytokines including IL-1β and TNF-α in the inflamed vessel prevent synthesis but promote MMP release, thus subverting tissue repair and favoring pathology (Siwik et al., 2000). Interestingly, after 7 days of infection, we observed an increase in tissue stiffness and a more brittle matrix despite the degradation of crosslinks. The mechanical properties of collagen are determined by the content, crosslinking, distribution and orientation of the individual fibers (Wu et al., 2003; Bayan et al., 2009). Our data shows that infected macrophages render the collagen fibers less uniform and thicker due to bundling of individual fibers, probably due to the exertion of mechanical forces during the migration of macrophages. Infection polarizes monocytes to an M2 state, which has been shown to interact with collagen through the formation of podosomes, and migrate in 2D and 3D collagen matrices (Cougoule et al., 2012). It has also been shown that sustained force generation by large, slow moving cells leads to increased structural organization of the matrix such as by aligning the fibrils parallel to the long axis of moving cells (Kim et al., 2006). Together, our data suggests that infected macrophages are capable of altering the structure and increasing the matrix stiffness. This may be another contributing role for *C. pneumoniae* infection to atherosclerosis, where the stiffness increases in diseased arteries by ∼1.5-fold compared to healthy arteries (Kohn et al., 2015). Of interest, macrophages infected with chlamydial antigens, though potent, was not as effective in establishing a pro-inflammatory microenvironment as that of live infection, highlighting the importance of sustained, low grade inflammation triggered by an intracellular organism on chronic diseases.

In summary, our results delineate possible contributing roles of *C. pneumoniae* infection in the pathogenesis of various stages of atherosclerosis. The 3D infection model has provided new insights not only into the secreted or biochemical factors but also into cell-matrix interactions or biophysical aspects. In a broader context, our results should provide framework for understanding the role of pathogens in atherosclerosis as there is mounting evidence that more than a single pathogen may be involved. This “Infectious Burden” can be a risk factor for atherosclerosis either directly by mechanistic link or indirectly by systemic inflammation or molecular mimicry, which can provide prophylactic or therapeutic options (Sessa et al., 2014).

## Author Contributions

SE and AR designed the study, analyzed the data, and wrote the manuscript. SE and SD performed the experiments.

## Conflict of Interest Statement

The authors declare that the research was conducted in the absence of any commercial or financial relationships that could be construed as a potential conflict of interest.

## Abbreviations

*C. pneumoniae*/Cpn: *Chlamydia pneumoniae*
Ex/Em: excitation/emission
HK-Cpn: Heat killed *Chlamydia pneumoniae*
LDL: Low density lipoprotein
MMP: Matrix metalloproteinases
