# Supplementary material for: Biophysical and Biochemical Outcomes of Chlamydia pneumoniae Infection Promotes Pro-atherogenic Matrix Microenvironment
Source: Front Microbiol. 2016 Aug 17;7:1287. doi: 10.3389/fmicb.2016.01287 (PMC4987350; doi:10.3389/fmicb.2016.01287)
Supplement: Supplementary file 1 [file Presentation_1.PDF]

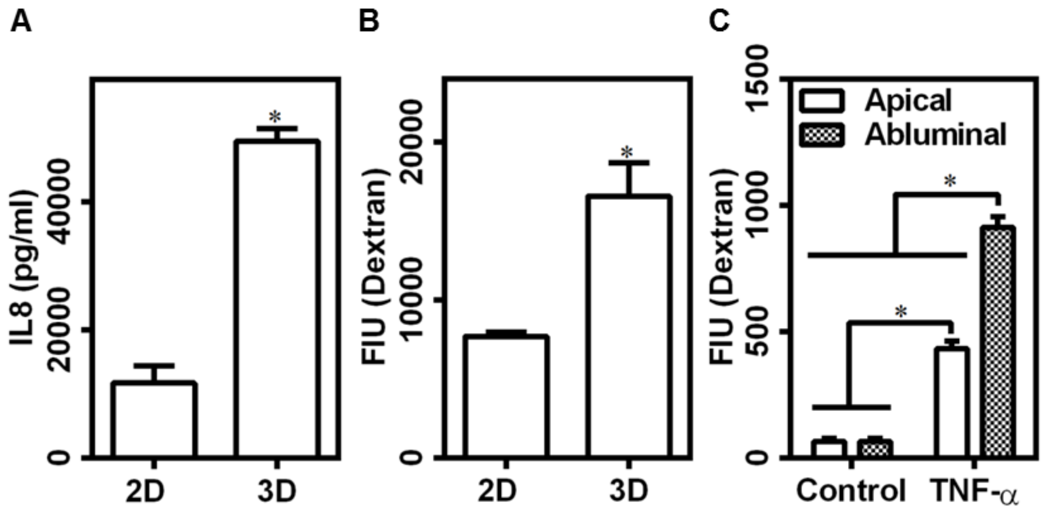

Figure S1. 3D environment and its importance: (A-B) Monocytes were embedded in 2mg/ml collagen or seeded as such in a 24 well plate. Post gelation, cells were supplemented with media and incubated for 24 hours. **(A)** The supernatants were collected form the samples and estimated for IL-8 by ELISA. The results are plotted as quantity of secreted protein as obtained from standard curve. **(B)** Trans-well containing confluent endothelial cells were placed in 24 well plate containing 2D and 3D gels with macrophages and incubated for 24 hours. Post incubation, media from upper side of trans-well was replaced with fluorescent dextran and the plate was incubated for 1 hour and supernatants were collected from bottom well. Fluorescent intensity from supernatant was measured using plate reader as an estimate of permeability. **(C)** Endothelial cells were grown to confluence in a trans-well. The media on either top or bottom of trans-well was replaced with media containing 0 or 20 ng/ml of TNF-α and incubated for 4 h. Post incubation, upper side of trans-well was replaced with media containing fluorescent dextran and the plate was incubated for 1 hour. Media from bottom of the trans-well was collected and fluorescence intensity was measured using plate reader. The results are mean ± SD of one representative experiment performed in triplicate, and the experiments were repeated three times. The * denote statistically significant change in the parameters between different groups, as calculated using Graph-Pad Prism ($P < 0.05$, ANOVA).

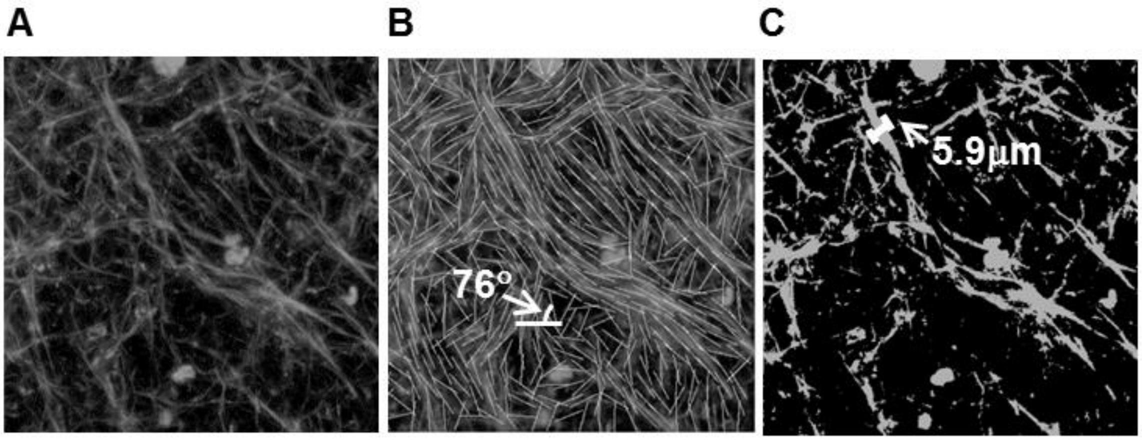

Figure S2. Analysis of matrix properties: After infection for 4 hours monocytes embedded in collagen with 10% FL – collagen and incubated for up to one week. Representative 2D overlay of a 3D confocal image in grey scale **(A)**, with calculated fiber angles as shown by red arrow **(B)** using Orientation J of Image J software and filament thickness as measured manually **(C)** by Image J software to calculate bundling (indicated by scale bar).

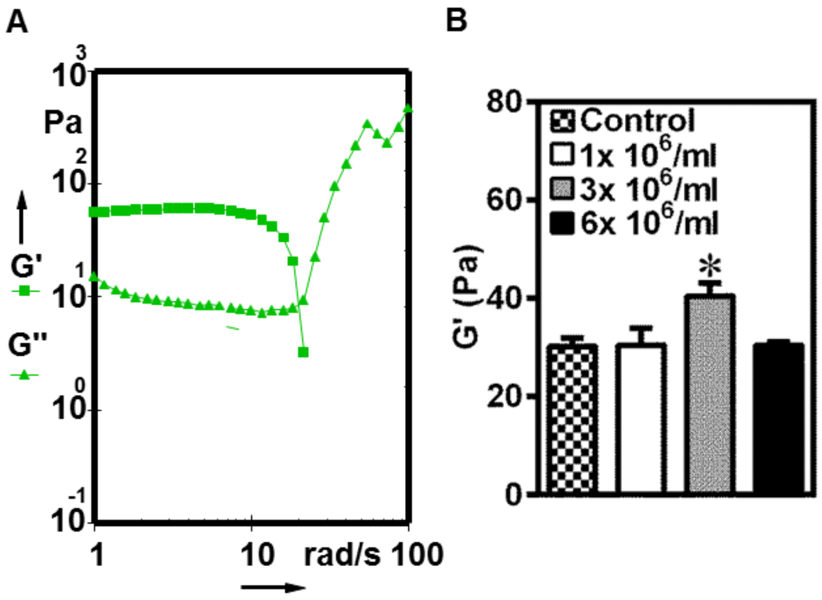

Figure S3. Cell concentration might affect the stiffness of the 3D collagen gels: Monocytes were embedded in 2 mg/ml of collagen at a concentration of up to 6 $x10^6$ cells/ml supplemented with media after gelation and incubated for 2 hours. **(A)** The gels were used to estimate stiffness using frequency sweep by dynamic shear rheometer. Representative frequency sweep is shown, and **(B)** stiffness of gels with different cell concentrations is shown. The results are mean ± SD of 1 experiment performed in triplicates and experiment was repeated 3 times. * denote statistically significant change in the parameters between different groups, as calculated using Graph-Pad Prism ($P <$ 0.05, ANOVA).
